# Self-supervised global context graph neural network for session-based recommendation

**DOI:** 10.7717/peerj-cs.1055

**Published:** 2022-07-28

**Authors:** Fei Chu, Caiyan Jia

**Affiliations:** School of Computer and Information Technology & Beijing Key Lab of Traffic Data Analysis and Mining, Beijing Jiaotong University, Beijing, China

**Keywords:** Self-supervised learning, Graph neural network, Session-based recommendation

## Abstract

Session-based recommendation (SBR) aims to recommend the next items based on anonymous behavior sequences over a short period of time. Compared with other recommendation paradigms, the information available in SBR is very limited. Therefore, capturing the item relations across sessions is crucial for SBR. Recently, many methods have been proposed to learn article transformation relationships over all sessions. Despite their success, these methods may enlarge the impact of noisy interactions and ignore the complex high-order relationship between non-adjacent items. In this study, we propose a self-supervised global context graph neural network (SGC-GNN) to model high-order transition relations between items over all sessions by using virtual context vectors, each of which connects to all items in a given session and enables to collect and propagation information beyond adjacent items. Moreover, to improve the robustness of the proposed model, we devise a contrastive self-supervised learning (SSL) module as an auxiliary task to jointly learn more robust representations of the items in sessions and train the model to fulfill the SBR task. Experimental results on three benchmark datasets demonstrate the superiority of our model over the state-of-the-art (SOTA) methods and validate the effectiveness of context vectors and the self-supervised module.

## Introduction

In the era of information explosion, recommendation systems (RS) play critical roles in various online commercial applications due to their success in addressing information overload by recommending useful context to users. Many existing recommendation approaches apply user profiles and long-term historical interactions to predict their preference, *e.g*., collaborative filtering (Sarwar et al., 2001), matrix factorization (Rendle et al., 2009), and deep learning based methods (He et al., 2017). However, in many real-world scenarios, such information may not exist. Consequently, session-based recommendation (SBR) has recently attracted more and more attention, which aims to predict the next interested item based on a given anonymous behavior sequence within a short period of time.

Early methods (Zimdars, Chickering & Meek, 2001) used Markov chains to predict the next item based on the previous clicked items in nature and have limited prediction accuracy due to the strong independence assumption. In recent years, with the development of deep learning, recurrent neural networks (RNNs) based methods (Hidasi et al., 2016) and graph neural networks (GNNs) based models (Wu et al., 2019) have been proposed and made great progress. RNNs-based models (Jannach & Ludewig, 2017; Li et al., 2017; Liu et al., 2018) can learn complicated and effective item-transitions patterns from users’ historical interactions. These models only consider the current session and ignore the complex high-order relationships between items in different sessions. However, individual session tends to be short, and the item transitions from other sessions may contain useful information about the current session. Unlike RNNs-based recommendation methods, GNNs-based methods model session sequences as graph-structured data. Pan et al. (2020) added a star node into a session graph to consider the non-adjacent items while ignoring cross-session information. Wang et al. (2020b) built a global graph over all sessions to learn global-level information, unified into the current session to improve the recommendation performance. Still, it may enlarge the impact of noisy interactions and does not consider the information from items without direct connections. Although multilayer GNNs are used to propagate information between items that are not directly connected, they can easily lead to over-smoothing (He et al., 2020).

Recently, self-supervised learning (SSL), especially contrastive learning (Hjelm et al., 2019), has become a hot research topic, which allows us to learn data representations from raw data with annotations. As a pioneer, Zhou et al. (2020b) used SSL to enhance the learning of data representations in a mutual information maximization manner for recommendations. Xia et al. (2021b) constructed two views to learn inter- and intra-session information and uses SSL to provide complementary information. However, how to choose the proper comparison perspective in contrastive learning is still a challenging problem in SBR on account of the information limitation of each session.

**Figure 1:**
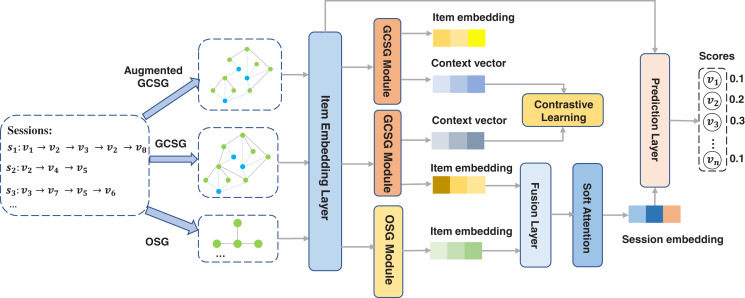
The workflow of SGC-GNN.

To address the above issues, we propose a self-supervised global context graph neural network (SGC-GNN) model for SBR. Figure 1 shows the workflow of the proposed SGC-GNN model. At first, we constructed a cross-session graph by adding a context vector for each session which can provide a natural way to pass information beyond adjacent items. We named the graph GCSG (Global Context Session Graph). In GCSG, we propose a global context graph neural network to model complex high-order relationships between items from the global level. We also constructed an Ongoing Session Graph (OSG) for each session. By modeling pairwise item transitions within the ongoing session, we can obtain the session-level item embedding. In addition, to obtain more robust item representations, we generated an augmented graph of GCSG and maximize the agreement of the context vectors of the same session in the original GCSG graph and the augmented graph, pushing away the agreement of the two context vectors with the other context vectors. We call this module a contrastive self-supervised learning module. Finally, SGC-GNN aggregated the learned item representations from global-level and session-level for SBR. By jointly optimizing the contrastive self-supervised task and the recommendation task, the model significantly improves the recommendation accuracy and enhances the robustness of the model against interaction noises.

The main contributions of this work are summarized as follows.

- To the best of our knowledge, this is the first work that adds context vectors to a global session graph to learn the relationships between item pairs that are not directly connected cross sessions.
- A novel supervised module is proposed to obtain more robust item representations in the global context graph.
- A unified scheme is used to combine the pairwise item-transition information in OSG and high-order relationships between adjacent and non-adjacent items in GCSG.
- Extensive experiments show that SGC-GNN has superiority over the SOTA baselines and achieves significant improvements on three real-world datasets.

## Related work

### Session-based recommendation

SBR aims to capture dynamic user preferences to provide more timely and accurate recommendations (Wang et al., 2021). Early SBR mainly used Markov decision process-based methods to capture the sequence signals in interactions. Wu et al. (2013) proposed a Personalized Markov Embedding (PME) model to embed both users and items into a Euclidean space in which the distance between users and items reflects the strength of their relationships. Le, Fang & Lauw (2016) developed a hidden Markov model to incorporating dynamic user-biased emission and context-biased transition for recommendation. With the development of deep learning, many methods take advantage of the powerful capabilities of deep neural networks to model the complex dependencies in interactions for recommendations. Wang et al. (2020a) proposed a method to incorporate dwell time in SBR and uses RNNs to predict the next item. Tan, Xu & Liu (2016) employed data augmentation techniques and considers temporal shifts in user behavior to improve the performance of SBR. Song, Elkahky & He (2016) employed an MLP layer to combine both long-term static and short-term temporal user preferences and trains model with a pre-train method.

In recent years, GNNs has developed rapidly. They are widely used in various fields (Zhou et al., 2020a), such as providing optimal bike station layouts in the area of decision support systems (Chen et al., 2021) and predicting traffic states (Zheng et al., 2020). In addition, some methods employ GNN to model the complex transitions within or between sessions which have shown promising results (Wang et al., 2021) in session recommendation. Wu et al. (2019) introduced GNN into SBRS firstly and achieves superior performance. Chen & Wong (2020) proposed a lossless encoding scheme to address the lossy session encoding problem and devises a shortcut graph attention layer to capture long-range dependencies. Qiu et al. (2019) proposed a weighted attention graph layer to learn the embedding of items and sessions for the next item recommendation. Wang et al. (2022) simulates users’ behavior patterns in the session without destroying the click order and highlights the critical preferences of users during the simulate process. Xu et al. (2019) dynamically constructed a graph structure for session sequences and uses the self-attention network and GNN to capture global dependencies and local short-term dependencies, respectively. Huang et al. (2021) developed a position-aware attention mechanism to learn item transitional regularities within individual sessions and proposed a graph-structured hierarchical relation encoder to capture the cross-session item transitions explicitly. Deng et al. (2022) decomposed the session-based recommendation workflow into two steps. They built a global graph over all session data, learn global item representations in an unsupervised manner, and later refine these representations in individual session graphs. Qiu et al. (2020) constructed a broadly connected session graph to exploit and incorporate cross-session information in the individual session’s representation learning. Although these studies demonstrate that GNN-based have achieved good performance, they construct graphs based only on the adjacency or sequential relationships of items, making it difficult to model complex higher-order relationships between items in different sessions effectively. Xia et al. (2021b) constructed a hypergraph to capture the high-order correlations among items, which works similarly to our approach. However, it ignored the critical sequential relationships of items in the session and introduces a lot of noise, which reduces the robustness of the model. Besides, Pan et al. (2020) added a star node into a session graph to consider the non-adjacent items, which inspired us to build nodes representing each session on the cross-session graph to learn higher-order relationships beyond adjacent items in different sessions.

### Self-supervised learning

Self-supervised learning, especially contrastive learning (Hjelm et al., 2019; Chen et al., 2020), designed to learn data representations from raw data with annotations, can learn user representations more robustly. Yao et al. (2021) proposed a multi-task SSL framework for large-scale item recommendations and devised a data augmentation method from the perspective of feature correlations. Wu et al. (2021) employed three types of data augmentation from different aspects and takes node self-discrimination as the self-supervised task to offer an auxiliary signal for representation learning. Xia et al. (2021a) learned session representations from the session view and the item view by a self-supervised graph co-training framework, which can iteratively select evolving pseudo-labels as informative self-supervision examples for each view to improve the performance of recommendation.

## Methods

### Problem statement

Let $V = \{v_1, v_2, \ldots, v_{|V|} \}$ denote the set consisting of all unique items involved in all sessions, where |*V*| is the number of all unique items. A session *S* can be represented by an item list $S = [v_1^s,v_2^s,...,v_m^s]$ ordered by timestamps, where $v_i^s \in V$ represents the *i*−th clicked item within the session *S*. The goal of SBR is to predict the next click item $v_{m + 1}^s$ for the session *S* given $v_1^s,v_2^s,...,v_m^s$.

### Ongoing session graph module

**Figure 2:**
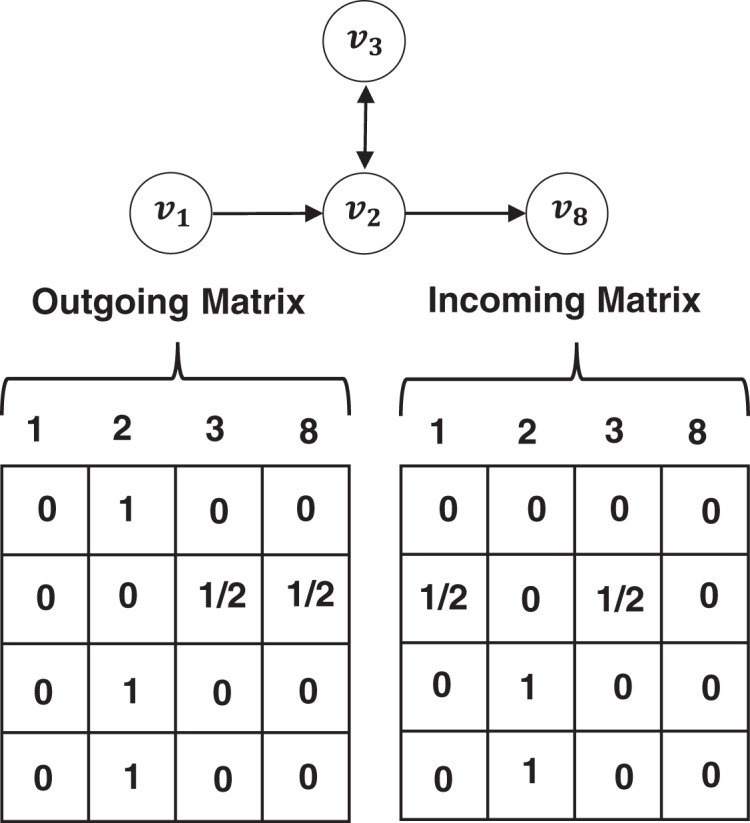
An example of OSG and its adjacent matrices.

An ongoing session graph (OSG) module aims to learn personalized item embedding by modeling sequential patterns in the current session. First, each session sequence *S* is modeled as a directed graph ${G_s} = ({{\rm {\cal {V}}}_s},{{\rm {\cal {E}}}_s})$. We name the graph OSG. Concretely, each node represents an item $v_i^s \in V$, each edge $(v_{i - 1}^s,v_i^s) \in {{\rm {\cal {E}}}_s}$ means that a user clicks item $v_i^s$ after $v_{i - 1}^s$ in the session *S*. The transition relationship between two items in the session can be represented by an incoming matrix and an outgoing matrix. For example, given a session *S* = [*v*_1_, *v*_2_, *v*_3_, *v*_2_, *v*_8_], let *G*_*s*_ denote its OSG graph, its incoming matrix **M**^*s*,*I*^ and outgoing matrix **M**^*s*,*O*^ are shown in Fig. 2. We concatenate the incoming matrix and the outgoing matrix to obtain a matrix **M** = Concat(**M**^*I*^, **M**^*O*^) for each session, which denotes how nodes in the OSG communicate with each other. We embed each item *v* ∈ *V* into a unified embedding space and the node vector ${\bf v} \in {{\rm {\mathbb R}}^d}$ indicates the latent vector of item *v*. Concretely, we generate a *d*-dimension embedding **v**_**i**_ for each unique item *v*_*i*_ in the session through an embedding layer. Then, we use the gated graph neural networks (GGNN) (Wu et al., 2019) to update each item embedding in the graph OSG, where the adjacency matrix **M**^*s*^ and the *l* − 1 layer embedding ${\bf v}_*^{s,l - 1}$ are used to update the embedding ${\bf v}_i^{s,l}$ of node *v*_*i*_ in *G*_*s*_ at layer *l* as follows.

(1) $$\eqalign{ {{\bf a}_i^{s,l}} & = {{\bf M}_{i:}^s{{[{\bf v}_1^{s,l - 1}, \ldots ,{\bf v}_n^{s,l - 1}]}^{\top}}{\bf W} + {\bf b}} \cr {{\bf z}_i^{s,l}} & = {\sigma ({{\bf W}_z}{\bf a}_i^{s,l} + {{\bf U}_z}{\bf v}_i^{s,l - 1})}\cr {{\bf r}_i^{s,l}} & = {\sigma ({{\bf W}_r}{\bf a}_i^{s,l} + {{\bf U}_r}{\bf v}_i^{s,l - 1})} \cr {\tilde {\bf v}_i^{s,l}} & = {\tanh ({{\bf W}_o}{\bf a}_i^{s,l} + {{\bf U}_o}({\bf r}_i^{s,l}{\rm \odot }{\bf v}_i^{s,l - 1}))}\cr {{\bf v}_i^{s,l}} & = {({\bf 1} - {\bf z}_i^{s,l}){\rm \odot }{\bf v}_i^{s,l - 1} + {\bf z}_i^{s,l}{\rm \odot }\tilde {\bf v}_i^{s,l}} \cr }$$

where ${\bf W},{{\bf W}_z},{{\bf W}_r},{{\bf W}_o} \in {{\rm {\mathbb R}}^{d \times 2d}}$ and ${{\bf U}_z},{{\bf U}_r},{{\bf U}_o} \in {{\rm {\mathbb R}}^{d \times d}}$ controls the weights, and ${\bf b} \in {{\rm {\mathbb R}}^d}$ is a bias vector. ${\bf z}_i^s,{\bf r}_i^s$ are the reset and the update gates, which decide what information to be preserved and discarded, respectively. *σ*(·) is the sigmoid function, and $\odot$ is the element-wise multiplication operator. $\tilde {\bf v}_i^s \in {{\rm {\mathbb R}}^d}$ represents the candidate state of node *v*_*i*_. And the final state ${\bf v}_i^s$ is the combination of the previous hidden state and the candidate state under the control of the update gate. However, a GNN with multiple layers is prone to over-fitting and over-smoothing. We utilize dropout (Srivastava et al., 2014) at each layer and highway network (Pan et al., 2020) to alleviate the problems. Concretely, we aggregate the output of the last layer of the module with the initial input as the final item representation in the following.

(2) $$\eqalign{ {\bf g} & = {\sigma ({{\bf W}_s}[{\bf v}_i^{s,0};{\bf v}_i^{s,l}])} \cr {{\bf v}_i^s} & = {{\bf gv}_i^{s,0} + ({\bf 1} - {\bf g}){\bf v}_i^{s,l}} \cr }$$

where ${{\bf W}_s} \in {{\rm {\mathbb R}}^{2d \times d}}$ are learnable parameters and $\left[ ; \right]$ is the concatenation operation.

### Global context session graph module

**Figure 3:**
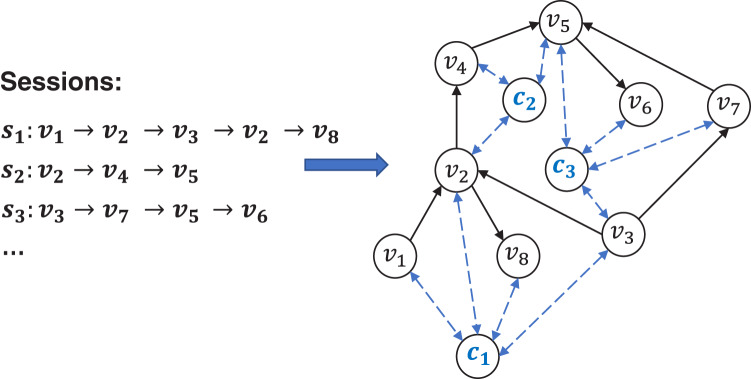
An example of GCSG consisting of three sessions, where $\bf{c}_1,\bf{c}_2,\bf{c}_3$ are the context vectors corresponding to sessions $s_1,s_2,s_3$, respectively.

The GCSG module aims to learn more powerful item embedding by modeling complex high-order relationships among items through context vectors over different sessions. Firstly, we formulate a cross-session item graph as $G = ({{\rm {\cal {V}}}_g},{{\rm {\cal {E}}}_g})$, in which nodes ${{\rm {\cal {V}}}_g}$ and edges ${{\rm {\cal {E}}}_g}$ are generated from historical sessions. Each session sequence *S* is viewed as a path which starts from $v_1^s$ and ends at $v_m^s$ in graph *G*. Unlike existing methods, we add a global representation for every session in the graph *G*, which is called a **master node** or a **context vector** (Gilmer et al., 2017; Battaglia et al., 2018). The context vector builds up a representation for the session as a whole and have a bidirectional edge to all other nodes in the session, providing a natural way to pass information between items that are not directly connected. We call the modified graph GCSG (global context session graph), formulate it as ${G_g} = ({{\rm {\cal {V}}}_g},{{\rm {\cal {E}}}_g},{{\rm {\cal {C}}}_g})$, where ${{\rm {\cal {C}}}_g}$ means context vectors. A simple illustration of GCSG is shown in Fig. 3.

#### Initialization

First, we initialize each item *v* ∈ *V* in a unified embedding space, yielding a representation of the item ${\bf v} \in {{\rm {\mathbb R}}^d}$ as mentioned in OSG module. In order to incorporate the sequential information into a context vector, we also add a learnable position embedding ${\bf p} \in {{\rm {\mathbb R}}^{d \times m}}$ to the item representation. More specifically, for each session $S = [v_1^s,v_2^s,...,v_m^s]$, we add **p** into ${\bf v} \in \{ {\bf v}_1^s,{\bf v}_2^s,...,{\bf v}_m^s\}$, *i.e*., **v**^*p*^ = **v** + **p**. We then take the representation of the last item *v*_*m*_ as the local embedding of the session *S*, *i.e*., **s**_*l*_ = **v**_*m*_. After this, we aggregate all node vectors of the session as the global preference embedding **s**_*g*_. Adopting the soft-attention mechanism to learn their priority, we hybrid the local and the global embedding **s**_*l*_ and **s**_*g*_ as below.

(3) $$\eqalign{ {{{\bf a}_i}} & = {{\bf W}_0^{\top}\sigma ({{\bf W}_1}{\bf v}_m^p + {{\bf W}_2}{\bf v}_i^p + {\bf b})} \cr {{{\bf s}_g}} & = {\sum\limits_{i = 1}^n {{\bf a}_i}{\bf v}_i^p} \cr {{{\bf s}_h}} & = {{{\bf W}_3}[{{\bf s}_l};{{\bf s}_g}]} \cr }$$

where ${{\bf W}_0} \in {{\rm {\mathbb R}}^d}$, ${{\bf W}_1},{{\bf W}_2} \in {{\rm {\mathbb R}}^{d \times d}}$ and ${{\bf W}_3} \in {{\rm {\mathbb R}}^{d \times 2d}}$ are the learnable parameters to control the weights of items, and ${\bf b} \in {{\rm {\mathbb R}}^d}$ is a bias vector. Finally, we use the hybrid embedding *s*_*h*_ as an initialization of the corresponding context vector **c**^*s*^, *i.e.*, **c**^*s*^ = **s**_*h*_. This strategy combines the long-term preference and the recent interests of the session, building up a good representation for the session as a whole.

#### Node updating

To learn high-order items transitions information from sessions, inspired by Pan et al. (2020), we alternately updated item embedding and context vectors on the global context session graph *G*_*g*_. For each node in *G*_*g*_, the information was collected and propagated from two sources: adjacent items and context vectors. First, we handle the graph *G*_*g*_ without considering context vectors in the same way we handle OSG. The construction of the incoming matrix and the outgoing matrix of the graph *G*_*g*_ is similar to the OSG. We also concatenate two matrices to get the matrix **M**. For each node *v*_*i*_ in graph *G*_*g*_ at layer *l*, we update node representation ${\bf v}_i^{g,l}$ from adjacent nodes across different sessions according to Eq. (1). We then added a dropout layer to alleviate over-fitting. Since items may appear in multiple sessions, each node in *G*_*g*_ may be connected to multiple context vectors. Suppose that the context vectors of the sessions containing node *v*_*i*_ form the set **c**_*i*_ = [**c**_*i*,1_, **c**_*i*,2_, …, **c**_*i*,*n*_], where *n* is the number of sessions containing the node *v*_*i*_. We first calculate the similarity *α*^*l*^_*i*,*j*_ of the node *v*_*i*_ and the context vector **c**_*i*,*j*_ in layer *l* with an attention mechanism as below.

(4) $$\alpha _{i,j}^l = \sigma ({{{{({{\bf{W}}_{q1}}{\bf{v}}_i^{g,l})}^ \top }{{\bf{W}}_{k1}}{\bf{c}}_{i,j}^{l - 1}} \over {\sqrt d }})$$

where ${{\bf W}_{q1}},{{\bf W}_{k1}} \in {{\rm {\mathbb R}}^{d \times d}}$ are the trainable parameters, $\sqrt d$ is used for scaling the coefficient, ${\bf v}_i^l$ and ${\bf c}_{i,j}^{l - 1}$ are the representation of node **v**_*i*_ at layer *l* and the context vector representation at layer *l* − 1, respectively. Then we obtain the representation of the node **v**_*i*_ from context vectors, which is a linear combination of ${\bf c}_j^l$ with the similarity $\alpha _{i,j}^l$ as a weight (*j* = 1, ⋯, *n*). After this, we calculate the level priority $\beta _i^l$ by performing a nonlinear map on the representation vectors ${\bf v}_i^{g,l}$ and ${\bf v}_i^{c,l}$ to balance the importance of the two vectors.

(5) $$\eqalign{ {} &{{\bf v}_i^{c,l} = \sum\limits_{j = 1}^m \alpha _{i,j}^l{\bf c}_j^l} \cr {} &{\beta _i^l = \sigma ({{\bf W}_4}[{\bf v}_i^{g,l};{\bf v}_i^{c,l}])} \cr }$$

where ${\bf v}_i^{g,l}$ is obtained from adjacent items and ${\bf v}_i^{c,l}$ is obtained from the context vectors at layer *l*, ${{\bf W}_4} \in {{\rm {\mathbb R}}^{2d \times d}}$ are learnable parameters. Then, applying a gate mechanism, we integrate the information from adjacent nodes and the related context vectors as follows.

(6) $$\eqalign{ {{\bf v}_i^l = ({\bf 1} - \beta _i^l){\bf v}_i^{g,l} + \beta _i^l{\bf v}_i^{c,l}}\cr }$$

where ${\bf v}_i^l$ is the representation of the node at layer *l*. Finally, we aggregated the output of the last layer and the initial input of the module similar to Eq. (2) to obtain the final item representation ${\bf v}_i^g$.

#### Context vector updating

For each context vector in the graph *G*_*g*_, we only use the representations of items in their corresponding session to obtain the representation of the context vector. First, we assigned different degrees of importance to each node *v*_*i*_ as below.

(7) $$\eqalign{ {\gamma _{j,i}^l = {\rm Softmax} \bigg(\displaystyle{{{{({{\bf W}_{k2}}{\bf v}_i^l)}^{\top}}{{\bf W}_{q2}}{\bf c}_j^{l - 1}} \over {\sqrt d }} \bigg)} \cr }$$

where ${{\bf W}_{q2}},{{\bf W}_{k2}} \in {{\rm {\mathbb R}}^{d \times d}}$ are the trainable parameters, $\gamma _{j,i}^l$ denotes the importance of the *i*-th item to *j*-th sessions at layer *l*. We then perform a linear combination of the item representations, and aggregate the updated context vector ${\bf c}_j^l$ and its *l* − 1 layer representation ${\bf c}_j^{l - 1}$ as follows.

(8) $$\eqalign{ {} &{{\bf c}_j^{{l}^{\prime}} = \sum\limits_{i = 1}^n \gamma _{j,i}^l{\bf v}_i^l}\cr {} &{{\varphi ^l} = \sigma ({{\bf W}_5}[{\bf c}_j^{{l}^{\prime}};{\bf c}_j^{l - 1}])} \cr {} &{{\bf c}_j^l = {\varphi ^l}{\bf c}_j^{l - 1} + ({\bf 1} - {\varphi ^l}){\bf c}_j^{{l}^{\prime}}} \cr }$$

where ${{\bf W}_5} \in {{\rm {\mathbb R}}^{d \times 2d}}$ are learnable parameters. In the same way as the final step of the node updating, we also use a highway network to combine the initialization of the context vector in the module and the output of the last layer to obtain the final representation **c**_*j*_.

### Self-supervised contrastive learning module

To improve the robustness of the model, we integrated self-supervised contrastive learning into the GCSG module. Since data augment methods are not the main concern of this study, we simply use the edge drop strategy to get an augmented graph of GCSG. Give a mini-batch of sessions $\{ {s_u}\} _{u = 1}^N$, we apply the edge drop on the GCSG *G*_*g*_ to obtain an augmented graph $G_g^{aug}$. We view the same session in the original GCSG and the augmented graph $({s_n},s_n^{aug})$ as a positive pair, and the other 2(*N* − 1) sessions in two graphs are considered as negative samples. For each session pair $({s_n},s_n^{aug})$, their updated context vectors are $({{\bf c}_n},{\bf c}_n^{aug})$. Since the context vectors can be viewed as an overall representation of the session, the updated context vectors obtained from the session pair can be naturally treated as a pair of positive samples. We adopted the InfoNCE loss (van den Oord, Li & Vinyals, 2019) of the context vectors in the two graphs as the optimization object defined below.

(9) $$\eqalign{ {{{\rm {\cal {L}}}_{\rm ssl}}({{\bf c}_n},{\bf c}_n^{aug}) = - \log \displaystyle{{\exp ({\rm sim}({{\bf c}_n},{\bf c}_n^{aug})\tau )} \over {\sum\nolimits_{m = 1}^{2N} \exp ({\rm sim}({{\bf c}_n},{\bf c}_m^{aug})/\tau )}}} \cr }$$

where sim(·, ·) is the similarity function, *e.g.*, dot-product, and *τ* is a hyper-parameter that controls the scaling. We use the self-supervised contrastive learning (SSL) loss on the context vector level instead of the item level to strengthen the robustness of the whole model.

### Session representation and prediction layer

For each item **v**_*j*_, we have two representations: One is obtained from the OSG module, and the other is obtained from the GCSG module, as mentioned before. Then, the final representation of the item is computed by sum pooling as follows:

(10) $${\bf v}_j^{\prime} = {\bf v}_j^s + \mu {\bf v}_j^g$$

where *μ* is a hyper-parameter to control the ratio of the representation learned from OSG module. Next, we calculate the representation of each session *s*_*i*_ in the same way as we initialize the context vectors by Eq. (3). We then obtain the final recommendation probability of the item as below.

(11) $${\hat {\bf y}_i} = {\rm Softmax}({\bf s}_i^{\top}{\bf v}_j^{\prime})$$

We used the cross-entropy of the prediction results $\hat {\bf y} = \{ {\hat {\bf y}_1}, \cdots ,{\hat {\bf y}_{|V|}}\}$ and the ground truth labels **y** as the main loss defined in the following:

(12) $${{\rm {\cal {L}}}_{rec}}(\hat {\bf y}) = - \sum\limits_{i = 1}^{|V|} {{\bf y}_i}\log ({\hat {\bf y}_i}) + (1 - {{\bf y}_i})\log ({\bf 1} - {\hat {\bf y}_i}).$$

We then combine the SSL loss with the recommendation loss to jointly optimize the recommendation task and the self-supervised task as follows:

(13) $${\rm {\cal {L}}} = {{\rm {\cal {L}}}_{\rm rec}} + \lambda {{\rm {\cal {L}}}_{\rm ssl}}$$

where *λ* is a hyper-parameter to control the ratio of contrastive SSL, the SSL loss is used as a regularization term to improve the effectiveness and the robustness of the whole model.

## Experiments

### Experimental settings

We conducted our experiments on three benchmark datasets, *Diginetica*, *Tmall* and *RetailRocket*. Following the previous work (Wang et al., 2020b), we filtered out sessions of length 1 and items that appear less than five times over all datasets. We set the sessions of the latest data (such as, the data of the last week) as the test data, the remaining historical data for training and validation. Furthermore, for a session *S* = [*v*_1_, *v*_2_, …, *v*_*m*_], we generated a series of sequences and labels ([*v*_1_], *v*_2_), ([*v*_1_, *v*_2_], *v*_3_), …, ([*v*_1_, *v*_2_, …, *v*_*m*−1_], *v*_*m*_), where [*v*_1_, *v*_2_, …, *v*_*m*−1_] is the generated sequence and *v*_*m*_ denotes the label of the sequence. The statistics of datasets are summarized in Table 1.

**Table 1:** Dataset statistics.

Dataset	Diginetica	Tmall	RetailRocket
#Training sessions	719,470	351,268	433,643
#Test sessions	60,858	25,898	15,132
#Items	43,097	40,728	36,968
#Average lengths	5.12	6.69	5.43

We adopted two widely used ranking-based metrics: *P*@*K* and *MRR*@*K* to evaluate the recommendation performance. A *P*@*K* score measures whether a target item is included in the top-K list of recommended items, and a *MRR*@*K* score considers the position of a target item in the list of recommended items. Higher metric values indicate better ranking accuracy. Moreover, we compare our model with the following session recommendation models to justify the effectiveness of our model.

- **FPMC** (Rendle, Freudenthaler & Schmidt-Thieme, 2010) combined the matrix factorization and Markov chain for recommendation.
- **GRU4Rec** (Hidasi et al., 2016) uses Gated Recurrent Unit (GRU) to model user sequences for session recommendation.
- **NARM** (Li et al., 2017) employs RNNs with attention mechanism to capture user’s main purpose.
- **STAMP** (Liu et al., 2018) utilizes attention layers to capture the general preference and the current interests of the last click of the current session.
- **SRGNN** (Wu et al., 2019) utilizes the gated graph neural networks to update item embeddings and uses the attention mechanism to compute session representations.
- **GCE-GNN** (Wang et al., 2020b) constructs two types of session graphs to capture local and global information.
- **SGNN-HN** (Pan et al., 2020) applies a star graph neural network to model transition relationship between items.
- ***S***^**2**^**-DHCN** (Xia et al., 2021b) constructs a hypergraph and a line graph to learn inter- and intra-session information and uses self-supervised learning to provide complementary information.
- **COTREC** (Xia et al., 2021a) construct two views to capture inter- and intra-session information and use a co-training strategy to iteratively select and evolve pseudo-labels as informative self-supervision examples.

The hyperparameters were selected on the validation set, which was randomly selected from the training set with a proportion of 10%. For a general setting, the embedding size is 256, the batch size is 1,024, and each session is truncated within a maximum length of 20. We adopt the Adam optimizer with an initial learning rate 1*e*^−3^ and a decay factor of 0.1 for three epochs. Moreover, the ***L***_**2**_ regularization is 10^−5^, the scale ratio *τ* is 0.2, the ratio of dropping edges is 0.3, and the ratio for all dropout layers is 0.1.

For the baseline models, we reported their results in their original papers directly, if available, since we use the same datasets and evaluation metrics. We use well-reproduced results from the literature for some models without public code data or using different datasets. We can find the results of FPMC, GRU4Rec, STAMP, and SRGNN models in Xia et al. (2021a, 2021b) which are also the baseline models in our study. In addition, since the public session recommendation datasets are usually split according to time, the distribution of samples at the latter positions in the training data is more similar to the test data than the samples at the former positions (Guo et al., 2022). Therefore, recommendation methods based on constructing graphs for individual sessions, such as SGNN-HN, without shuffling the model will fit better. However, for methods that build graphs based on multiple sessions, not shuffling can lead to label leakage during testing. For fairness, we rerun the source code of the SGNN-HN model by shuffling the training datasets. Since we could not find the result of GCE-GNN on the “RetailRocket” dataset, we reran GCE-GNN and SGNN-HN and adjusted the hyperparameters of the models by grid search, and reported the average results on 10 random seeds.

### Results and analysis

The experimental results of overall performance are present in Table 2. In Table 2, our model (SGC-GNN) consistently achieved a good performance (statistically significant) on three datasets with both evaluation metrics, verifying our model’s superiority. From the results, we can draw the following conclusions.

**Table 2:** Performances of all comparison methods on three datasets.

Method	RetailRocket				Tmall				Diginetica			
	P@10	M@10	P@20	M@20	P@10	M@10	P@20	M@20	P@10	M@10	P@20	M@20
FPMC	25.99	13.38	32.37	13.82	13.10	7.12	16.06	7.32	15.43	6.20	22.14	6.66
GRU4Rec	38.35	23.27	44.01	23.67	9.47	5.78	10.93	5.89	17.93	7.73	30.79	8.22
NARM	42.07	50.22	24.88	24.59	19.17	10.42	23.30	10.70	35.44	15.13	48.32	16.00
STAMP	42.95	50.96	24.61	25.17	22.63	13.12	26.47	13.36	33.98	14.26	46.62	15.13
SR-GNN	43.21	26.07	50.32	26.57	23.41	13.45	27.57	13.72	38.42	16.89	51.26	17.78
GCE-GNN	47.83	28.07	55.82	28.63	28.01	15.08	33.42	15.42	41.16	18.15	54.22	19.04
SGNN-HN	48.88	29.27	56.70	29.81	29.97	16.64	36.30	17.04	40.82	17.95	54.19	18.87
*S*^2^-DHCN	46.15	26.85	53.66	27.30	26.22	14.60	31.42	15.05	40.21	17.59	53.66	18.51
COTREC	48.61	29.46	56.17	29.97	30.62	17.65	36.35	18.04	41.88	18.16	54.18	19.07
SGC-GNN	**50.56** *	**30.14** *	**58.77** *	**30.71** *	**36.09** *	**20.83** *	**41.62** *	**21.19** *	**41.97** *	**18.58** *	**55.49** *	**19.33** *

**Notes:**

Best performing method is shown in bold.

The second best performing method is shown with an underline.

* Indicates the statistical significance for *p* < 0.01 compared to the best baseline method with paired *t*-test.

- The methods (*i.e*., GRU4REC, NARM, STAMP, SR-GNN) that take into account temporal information achieve better results than the traditional methods (*i.e*., FPMC). It demonstrates the importance of sequential effects for SBR. Moreover, all methods using deep learning techniques perform well, which indicates the powerful ability of deep learning models in SBR.
- Graph-based methods all achieve better results than the RNN-based methods, demonstrating the ability of GNNs to model session data. Besides, the methods (*i.e*., GCE-GNN, *S*^2^-DHCN, COTREC) which capture different levels (inter- and intra- level) of information achieve better results than SRGNN, which only consider intra-session information. It demonstrates the usefulness of different levels of information for predicting the user’s intention in SBR.
- Our proposed model SGC-GNN outperforms all the baselines on all datasets. In particular, on both Tmall and RetailRocket, our model achieves significant improvement compared to the other methods, showing the effectiveness of the proposed model. The improvement of the SGC-GNN model against the baselines mainly comes from three aspects. The first one is the proposed global context session graph (GCSG). By introducing a global context vector as representative nodes for each session on the cross-session graph, GCSG can help learn the relationship between every two items in a session and the high-order relationships between non-adjacent items in different sessions. Thus, each node can obtain much information and learn richer node representations. The second is using a unified model to improve the recommendation performance of the current session. Moreover, the last one is using self-supervised contrastive learning to improve the robustness of the model. At the same time, other cross-session approaches suffer from reduced model robustness due to the large amount of noisy information introduced by the construction of cross-session graphs.

#### Ablation study

To investigate the contributions of each component in SGC-GNN, we developed three variants: **GNN-NC**, **SGC-GNN-NL** and **GC-GNN**. In GNN-NC, we removed context vectors and the self-supervised learning (SSL) module. In SGC-GNN-NL, we removed the session-level graph OSG. GC-GNN represents the version without the SSL module. We show the results of these variants compared to full GCS-GNN in Table 3 on two datasets *Tmall* and *RetailRocket*. We can observe that when the global context vectors are removed, there is a significant decrease in both metrics. It shows that the global context vectors are very helpful to performance improvement. Also, the SSL module effectively improves the model’s performance. Without the SSL module, the two metrics have different degrees of decrease on both datasets.

**Table 3:** Ablation experiments.

Method	Tmall		RetailRocket	
	P@20	MRR@20	P@20	MRR@20
GNN-NC	38.09	18.76	58.28	30.56
GC-GNN	40.39	20.67	58.76	30.62
SGC-GNN-NL	41.45	21.17	58.70	30.46
SGC-GNN	41.62	21.19	58.77	30.71

#### Impact of initialization of context vectors

**Figure 4:**
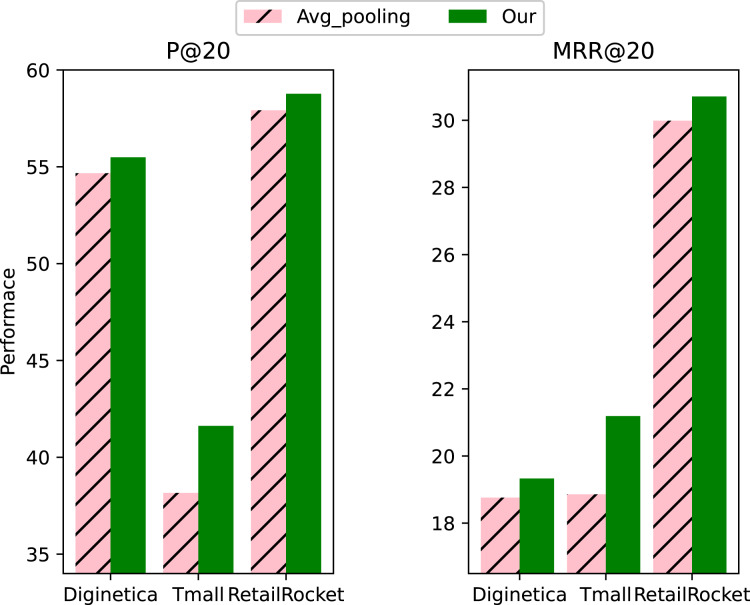
Impact of initialization method of context vectors.

To investigate the effectiveness of the initialization method of context vectors, we compared it with the average pooling initialization. From the results in Fig. 4, we can see that the initialization method we used for context vectors works significantly better than the average pooling initialization, proving the effectiveness of our proposed initialization method. Our proposed context vector initialization method assigned different weights to each item in the session instead of simply averaging the aggregates. The learned representation is more effective as a global representation of the session.

#### Impact of self-supervised learning

**Figure 5:**
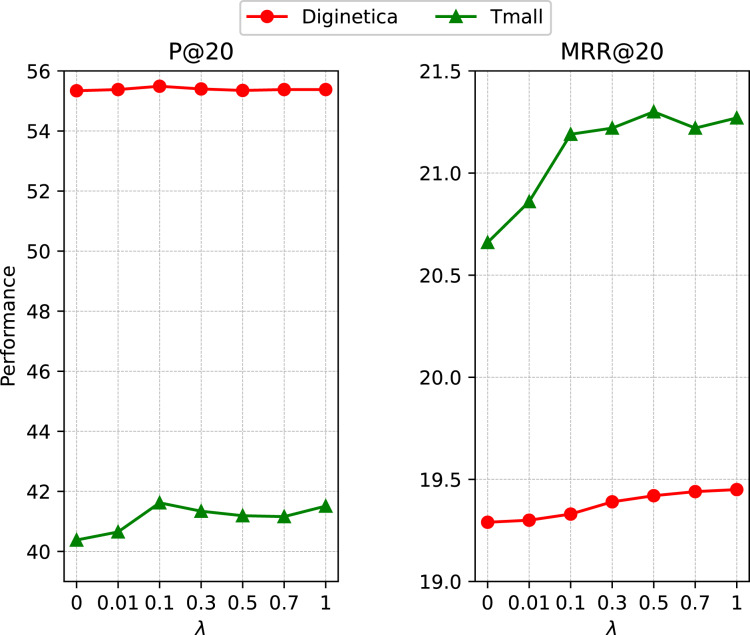
Impact of the ratio of self-supervised learning loss.

We introduced a hyper-parameter *λ* to control the magnitude of self-supervised learning. To investigate its influence, we reported the performance with a set of representative *λ* values in {0, 0.01, 0.1, 0.3, 0.5, 0.7, 1} on *Tmall* and *Diginetica*. According to the results presented in Fig. 5, the recommendation task achieves good gains when jointly optimized with the SSL task. The proposed self-supervised contrastive learning module performs data augmentation on the cross-session graph and then imposes InfoNCE loss on the generated global context vectors, enabling the model to learn more essential features and make it more robust.

#### Impact of OSG module

**Figure 6:**
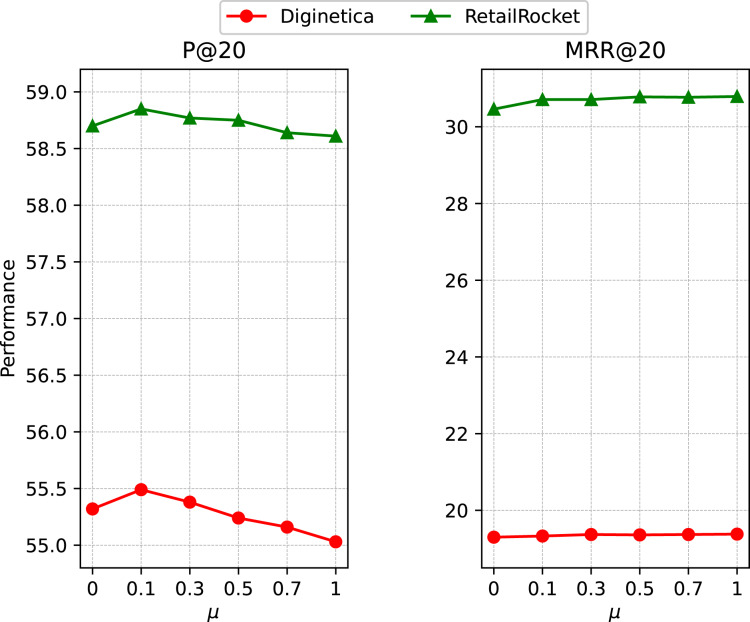
Impact of the ratio of OSG module.

To investigate the impact of the ratio of OSG module, we report the performance with a set of representative *μ* values in {0, 0.1, 0.3, 0.5, 0.7, 1} on *RetailRocket* and *Diginetica*. From the results in Fig. 6, we can see the effectiveness of the OSG module, while the model achieves better performance when the ratio *μ* takes a small value. With a unified model, we can aggregate the item embedding learned from the global and session levels to improve the current session’s recommendation performance.

#### Efficiency

We evaluated the training efficiency of SGC-GCN and its variant GC-GNN. Since COTREC and DHCN models require an older version of the environment to run, we chose to compare the efficiency with the SRGNN, SGNN-HN, and GCE-GNN methods. For a fair comparison, we set the batch size to 100 and the hidden size to 100 for all methods instead of putting them to 1,024 and 256, respectively, because large batch size can cause GCE-GNN to run out of memory. All experiments were conducted on a single Nvidia RTX A4000 GPU and the same computation environment. All methods were trained with 10 epochs, and we reported the average training time per epoch. The results are shown in Table 4.

**Table 4:** Performances of average training time(s) per epoch.

Method	Tmall	RetailRocket	Diginetica
SRGNN	282	1,519	606
SGNN-HN	283	339	559
GCE-GNN	169	–	953
GC-GNN	343	448	604
SGC-GNN	447	549	766

From Table 4, we can observe that SGC-GCN performs worse than other methods on the Tmall dataset, but on the Diginetica dataset, our model has about the same time as other methods. Both SGC-GNN and GCE-GNN models build larger session graphs containing multiple session information. Still, the GCE-GNN has a more complex structure, making it suffer from the out-of-memory problem when performing on RetailRocket on our RTX A4000 GPU. Moreover, if we consider the model GC-GNN that removes the self-supervised contrastive learning, its time consumption is similar to that of SRGNN and SGNN-HN. However, the difference in the training time of SGC-GNN is acceptable considering the performance improvement.

## Conclusion

Existing graph-based recommendation methods have difficulty modeling the relationship between non-adjacent items and introduce noisy information in constructing the global graph, which reduces the robustness of the model. In this study, we proposed a self-supervised global context graph neural network model SGC-GNN to solve this problem. In the model, we used global context vectors as a bridge for passing information between non-adjacent items in different sessions, allowing the model to learn a richer representation of nodes. At the same time, to address the problem of introducing a large amount of noisy information and thus reducing the robustness of the model due to the construction of cross-session graphs, we designed a self-supervised contrastive learning module that effectively improves the robustness of the model by augmenting the data and imposing InfoNCE loss on the global context vectors as an auxiliary loss of the model. Finally, we combined session-level information with global-level information through a unified model to enhance the feature presentations of items. Experimental results and analysis demonstrate the superiority of the proposed model.

## Supplemental Information

10.7717/peerj-cs.10551055/supp-1 Supplemental Information 1 The dataset and code.

## Additional Information and Declarations

### Data Availability

The following information was supplied regarding data availability: The data and code are available in the Supplemental Files.
